# A three-step, “brute-force” approach toward optimized affine spatial normalization

**DOI:** 10.3389/fncom.2024.1367148

**Published:** 2024-07-08

**Authors:** Marko Wilke

**Affiliations:** ^1^Department of Pediatric Neurology and Developmental Medicine, Children’s Hospital, University of Tübingen, Tübingen, Germany; ^2^Experimental Pediatric Neuroimaging, Children’s Hospital and Department of Neuroradiology, University of Tübingen, Tübingen, Germany

**Keywords:** spatial normalization, affine parameters, structural MRI, iterative approach, brute-force

## Abstract

The first step in spatial normalization of magnetic resonance (MR) images commonly is an affine transformation, which may be vulnerable to image imperfections (such as inhomogeneities or “unusual” heads). Additionally, common software solutions use internal starting estimates to allow for a more efficient computation, which may pose a problem in datasets not conforming to these assumptions (such as those from children). In this technical note, three main questions were addressed: one, does the affine spatial normalization step implemented in SPM12 benefit from an initial inhomogeneity correction. Two, does using a complexity-reduced image version improve robustness when matching “unusual” images. And three, can a blind “brute-force” application of a wide range of parameter combinations improve the affine fit for unusual datasets in particular. A large database of 2081 image datasets was used, covering the full age range from birth to old age. All analyses were performed in Matlab. Results demonstrate that an initial removal of image inhomogeneities improved the affine fit particularly when more inhomogeneity was present. Further, using a complexity-reduced input image also improved the affine fit and was beneficial in younger children in particular. Finally, blindly exploring a very wide parameter space resulted in a better fit for the vast majority of subjects, but again particularly so in infants and young children. In summary, the suggested modifications were shown to improve the affine transformation in the large majority of datasets in general, and in children in particular. The changes can easily be implemented into SPM12.

## 1 Introduction

Spatial normalization is commonly used to compare magnetic resonance (MR) images between subjects. Usually, the first step is an affine procedure, correcting for global differences in orientation and brain size; this is then commonly followed by regional feature matching using non-linear approaches (Friston et al., 1995; Wilke et al., 2002; Ashburner and Friston, 2005). As the ensuing non-linear deformations and/or tissue segmentations depend and rely on a good initial fit, this step is an important part in the complete processing stream. In a classical paper, Ashburner et al. (1997) developed a first set of reference values for the initial affine part of this process within the popular SPM software package, from a population of 51 subjects. The mean scaling factor for each dimension were determined and then used as starting estimates for the affine procedure. In the current version of the software (SPM12, 2023), these starting estimates are now based on 227 subjects (as detailed in the corresponding function spm_affine_priors, V7377). They are used to inform the initial guess for the affine spatial normalization procedure, which is then iteratively modified to achieve the best overlap between the individual image and the reference image. To this effect, the procedure is using a mutual information goodness-of-fit criterion (D’Agostino et al., 2004). Within the SPM12 software, this particular set of starting estimates is referred to as “MNI regularization” and is used by default. This procedure is computationally highly efficient: providing a mean and a standard deviation for each parameter-to-be-optimized substantially narrows the search space, avoids excessive values, and consequently, saves time (Ashburner et al., 1997). However, while providing reasonable starting estimates for most datasets, it may be not optimal for unusual datasets in general, and in three specific settings in particular.

One, a (technical) issue with spatial normalization in general and the affine part in particular is that MR images are usually corrupted by smoothly-varying intensity fluctuations induced by inhomogeneities of the magnetic field (Bernstein et al., 2006). Such bias fields are usually modelled, and removed, during processing (Vovk et al., 2007; Weiskopf et al., 2011; Ganzetti et al., 2016). Crucially, this often happens only after the initial affine registration: in SPM, for example, non-linear spatial normalization, tissue segmentation, and bias field estimation are jointly achieved in one iterative process (Ashburner and Friston, 2005), but only after the initial affine step. Similar approaches are used by other packages as well, such as FAST, or FreeSurfer (Despotović et al., 2015). Hence, the initial affine part of spatial normalization is performed on uncorrected (and therefore, still inhomogeneous) images. Consequently, such inhomogeneity-induced intensity variations might end up being “matched” to true tissue boundaries in the template image (Vovk et al., 2007; Wilke et al., 2017; SPM Manual, 2023), and misregistration may ensue.

Two, image registration may fail if the image-to-be-registered and the target (template) image are too different (Fookes and Bennamoun, 2002). Intensity-based approaches in particular may fail if the registration process encounters a local maximum, which may then be “matched” instead of finding a more appropriate global solution (Yokoi et al., 2004; Gao et al., 2008). Such issues may be avoided by reducing the input image complexity (Ince et al., 2017), for example by simply dividing an image into “head” and “background.” This may be beneficial as less features in a complexity-reduced image may be matched more robustly.

Three, there are issues with the starting estimates/prior information approach when it comes to images that do not conform to the assumptions underlying it. In this context, this most notably includes the brains of children, which are not only significantly smaller than the brains of adults (Mennes et al., 2014; Martini et al., 2018; Barkovich et al., 2019) but also change rapidly (with head circumference increasing by about 150% in the first 3 years of life alone; CDC, 2021). While this difference in overall brain size was suggested to be less of an issue as of about 6 years of age (Huttenlocher, 1979; Wilke et al., 2002; Wilke, 2014), it must be expected to be of high relevance in younger children. Also, differences in shape and volumetric as well as spatial relations may persist for much longer, and may also be relevant when investigating ethnicities that did not contribute to the default starting estimates (Uchiyama et al., 2013; Xie et al., 2015; Dong et al., 2020). As a normal distribution is used to describe the starting estimates (cf. the Gaussian curve fitted to the distribution in Figure 3 of Ashburner et al., 1997), this method will inherently favor datapoints within (and punish those outside) the thus-defined normal range. While a “no regularization” option is also available, this only “makes and provides no assumptions” about meaningful starting estimates in so far as that a scaling of 1 is assumed in each dimension. Therefore, “unusual brains” may not be treated appropriately by this approach.

**Figure 1:**
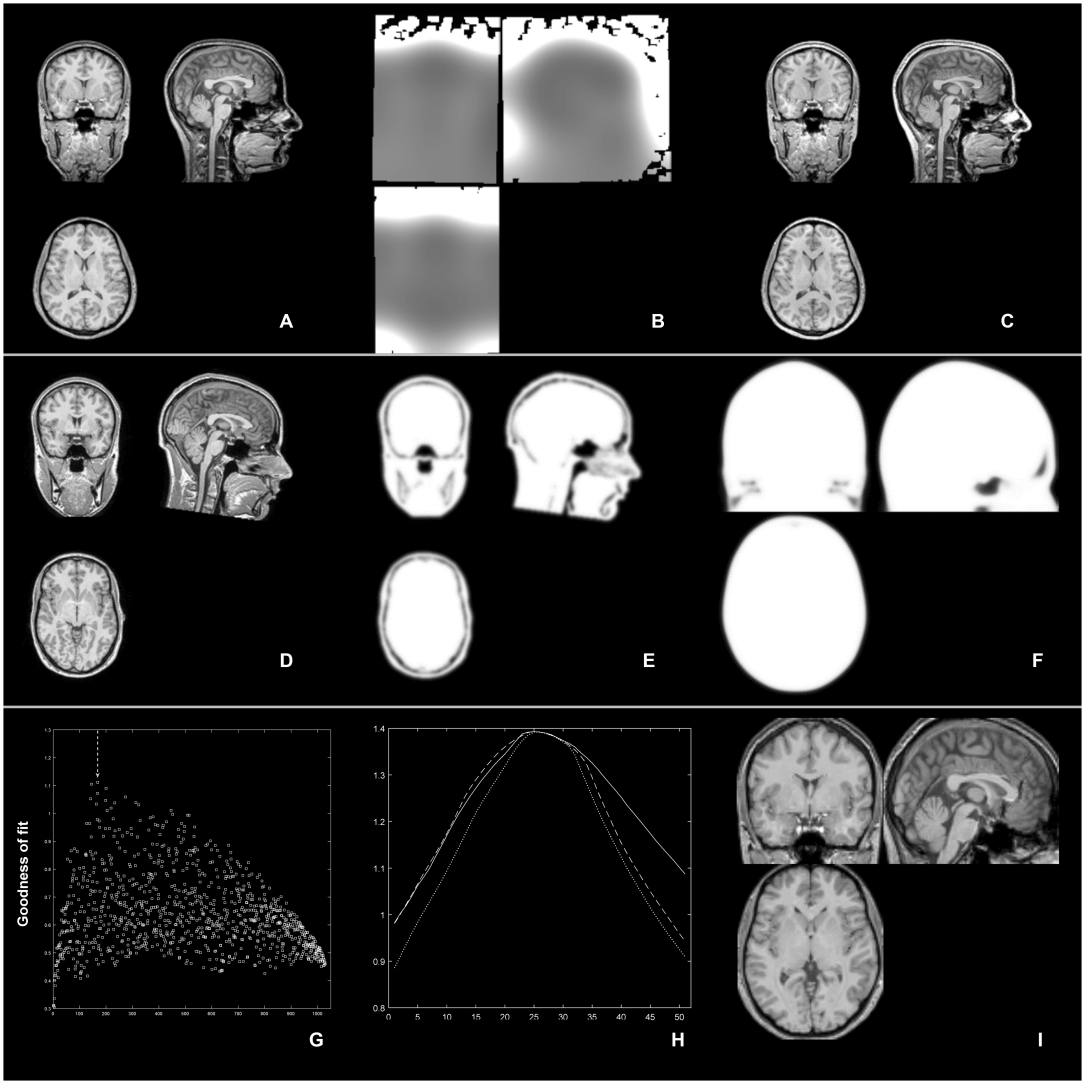
Overview of the 3 step approach: top row (inhomogeneity removal): from an MR image **(A)**, image inhomogeneities are removed **(B)** and the bias-corrected image **(C)** is then used to derive the affine transformation parameters; middle row (complexity-reduced image): an MR image **(D)** is binarized and smoothed (and thus, complexity-reduced; **E**) and is then matched to a similarly-reduced template **(F)**; bottom row (iterative processing): from an initial, unbiased exploration of the whole search space, the best of 1,000 combination is identified (highest “goodness of fit” value in **G**, arrow); thereupon, each scaling parameter (in *X*, *Y*, and *Z*) is further refined in 51 smaller steps **(H)** to finally yield an optimized parameter set for affine spatial normalization **(I)**.

The aim of this manuscript, therefore, is threefold: one, to assess the utility of performing affine spatial normalization on already bias-corrected images. For this aim, the hypothesis is that this will result in a better match to the template image as a function of image inhomogeneity. Two, to implement a robust initial matching step of a complexity-reduced input image to decrease the vulnerability toward local maxima. For this aim, the hypothesis is that an initial coarse match will lead to a better final match for unusual images in particular. Three, to develop and evaluate a new approach to assess and apply “broader” starting estimates in an unbiased, “brute force” fashion, which first requires the determination of a wide range of possibly normal scaling factors. The hypothesis is that blindly exploring this (as compared to “adults only”) widened parameter space will result in a better affine matching for “unusual” (e.g., pediatric) brain MR images in particular. An overview of the three steps is provided in Figure 1.

## 2 Subjects and methods

### 2.1 Subjects

A dataset of a total of 2081 images was compiled from open data repositories, covering the age range from 0 to 1,036 months (0–86 years). As before (Wilke et al., 2017), data from four sources was used, namely the NIH Study of Normal Brain Development (NIH, 2021; *n* = 500), the Cincinnati MR Imaging of Neurodevelopment study (C-MIND, 2023; *n* = 235), the 1,000 functional connectome study (fCONN, 2023; *n* = 783) and the Information eXtraction from Images study (IXI, 2023; *n* = 563). Other than in that previous publication, all subjects were used here, including those below 1 year and those above 75 years of age. For a broad categorization, minors were subdivided into young (group YM, <6 years), middle (group MM, ≥6, <12 years) and old (group OM, ≥12, <18 years) subgroups, as opposed to adults (group AD, ≥18 years). Demographic details of all subjects can be obtained from Table 1. More details on the individual datasets used are also available in the Supplementary material.

**Table 1:** Overview of demographic details of the whole cohort and the four subgroups, including the proportion of 3T images.

	Minors			Adults (AD)	Whole group
	Young (YM)	Middle (MM)	Old (OM)		
*n*	221	323	200	1,337	2081
Male	112 (50.6%)	144 (44.6%)	110 (55%)	600 (44.9%)	966 (46.4%)
3T	93 (42.1%)	103 (31.8%)	70 (35%)	1,121 (83.8%)	1,387 (66.6%)

### 2.2 MRI data preparation

Whole-brain 3D-T1-datasets from both 1.5T and 3T MR scanners were used. To minimize effects of initial positions and voxel sizes, all images were aligned with a T1-template using an automated rigid-body approach (Larroque, 2021), and the orientation of each image was manually adapted when necessary. Thereafter, images were resliced to a spatial resolution of 1.5 mm × 1.5 mm × 1.5 mm voxel size in native space using a 7th degree B-spline interpolation approach (Unser, 1999). As an unnecessary large field of view impairs data processing based on similarity measures (such as spatial normalization; Ou et al., 2018), images were automatically cropped to remove ~90% of empty slices. All processing and analysis steps were done employing functionality available within SPM12 (v7771, Wellcome Department of Imaging Neuroscience, University College London, United Kingdom) or using custom scripts and functions, running within Matlab (The Mathworks, Natick, United States). Visualization was achieved using violin plots (Bechtold, 2016) as these provide more information about the distribution of values.

### 2.3 Image registration metrics (mutual information criterion and Dice index)

To assess image registration performance, two metrics were used. For one, the magnitude of the final mutual information similarity criterion (D’Agostino et al., 2004) was used. This goodness-of-fit parameter is internally derived and used to optimize the overlap between the input and the reference image; a higher value means “better overlap.” This parameter is used by the affine registration algorithm to define “registration accuracy”; as such, it is a logical choice to assess if the one or the other approach performs “better.”

However, there are concerns regarding the reliability of this parameter (Rohlfing, 2012), and a high values does not in and of itself necessarily indicate an optimal overlap. Further, a “better” affine registration does not necessarily mean that the tissue segmentation resulting from this is also “better.” Further, only using the parameter that is optimized in the process as the sole outcome criterion for success would render the procedure open to criticism regarding circularity (Kriegeskorte et al., 2009). Hence, as an independent measure of actual tissue overlap, the Dice similarity coefficient (DSC; Dice, 1945) was also calculated. The DSC is a comprehensive indicator of overlap between two images and is commonly used to assess results of tissue segmentation (Eelbode et al., 2020). It is calculated taking into account the true positives (TP), the false positives (FP) and the false negatives (FN) to calculate DSC = 2 × TP/(2 × TP + FP + FN). The DSC ranges from 0 to 1, with 0 indicating no and 1 indicating perfect correspondence. To this effect, gray matter maps were obtained using unified segmentation (Ashburner and Friston, 2005) using either the traditional or the new approaches (see below) to initialize affine matching. Here, true positives would be voxels identified as gray matter in the map-under-study as well as in the reference map, false positives would be voxels identified in the map-under-study but not in the reference map, and false negatives would be voxels erroneously not identified in the map-under-study while they are present in the reference map. To assess only the effect of affine spatial normalization, later non-linear matching was disabled (by setting the respective regularization parameters to Inf). Of note, to compare two tissue partitions using the Dice index, binary values are required. Hence, tissue maps were binarized at a threshold of 0.1, as recommended for gray matter voxel-based morphometry studies (Gaser, 2023). These binary maps were then compared with the gray matter tissue prior to which they were normalized, resulting in one value per subject, per step.

#### 2.3.1 Objective 1: pre-affine inhomogeneity correction step

Within SPM’s unified segmentation approach, inhomogeneity correction, tissue segmentation, and non-linear spatial normalization are iteratively performed in succession within one comprehensive generative model (Ashburner and Friston, 2005). Consequently, the initial affine registration (the first step in spatial normalization) is derived from uncorrected images. To circumvent this, a simple two-step procedure was implemented similar to the one used before (Wilke et al., 2017). Here, images are initially “segmented” (omitting the affine registration, and hence, the inhomogeneity vulnerability inherent in it) to allow for the estimation and removal of the bias field using SPM’s own approach (Ashburner and Friston, 2005). As bias correction was the main aim, bias field regularization was set to “very light” (instead of the default “light”), and the “bias FWHM” cutoff option was set to “50 mm” (instead of the default “60 mm”). These slightly more liberal settings were chosen as completely disabling regularization would lead to unrealistic results (as then, true tissue boundaries would be “recognized” as inhomogeneity, and labeled as such). From this step, only the bias-corrected image was written out in native space. This was then used to determine the affine spatial normalization parameters, under the assumption that less inhomogeneity in the input image will allow a better fit to the tissue priors (as these of course also are inhomogeneity-corrected). Of note, the temporary bias-corrected image was only used to obtain the affine parameters, which were then applied to the original image. This ensures that “unified segmentation” is ultimately performed on the (inhomogeneous, but “optimally affine registered”) original image, to allow regular bias field modeling as part of the integrative model (Ashburner and Friston, 2005). This avoids the dangers of overcorrection (by applying bias correction twice) as well as of violating assumptions inherent in the bias-field estimation.

##### 2.3.1.1 Experiment 1a

To assess the utility of this approach, the affine matching using both the original as well as the bias-corrected images was compared using both image registration metrics described above. All other settings were left at their respective defaults. The number of images benefitting from this approach (in terms of higher values for the mutual information criterion and the DICE-index, respectively) and the actual values of both criteria were compared, for each age group.

##### 2.3.1.2 Experiment 1b

To test the assumption that the effect of such an approach is a function of the amount of inhomogeneity in a given image, the bias field itself was obtained by dividing the bias-corrected by the original image; here, all deviations from 1 reflect the effect of inhomogeneity correction. “Total inhomogeneity” was therefore determined by summing the voxelwise absolute magnitudes of the bias field. This value (in arbitrary units) was then compared between images benefitting and not benefitting from this approach.

#### 2.3.2 Objective 2: robust fitting

Tissue matching within SPM’s unified segmentation approach is based on a six-class tissue prior, containing gray and white matter, cerebrospinal fluid (CSF), bone, soft tissue, and background (for an illustration, see Wilke et al., 2017). Unless stated otherwise, the default tissue prior coming with SPM12 (in …\TPM\TPM.nii) was used for all analyses. The idea here was to reduce the complexity of the input image [and thus, of the matching procedure (Ince et al., 2017)] by binarizing it, with the aim to make the procedure more robust. Dividing the image into only two categories (“head” and “background”) and matching it to a similarly complexity-reduced tissue prior should avoid local minima as fewer solutions to the thus-simplified problem must be expected (Yokoi et al., 2004).

##### 2.3.2.1 Experiment 2

For each image under study, identifying the head (and separating it from background) was achieved by finding the mean value of all non-zero voxels and then isolating the largest cluster of above-average values (similar to an approach used in CAT12; Gaser, 2023). Small holes (default: up to 100 mL) were filled, and the binary image was finally smoothed by a Gaussian filter (FWHM = 4 mm) to yield a smoother, complexity-reduced source image. The target image (in the context of the SPM approach to affine registration, the standard 6-class tissue prior) was similarly reduced by summing all brain and head classes into one and contrasting it with the background class to yield a new “2-class prior.” This 2-class prior was then used to iteratively match the complexity-reduced input image to, using the standard spm functions. As done above, the effect of using the thus-derived parameters on the original image registration was assessed using the goodness-of-fit parameter and the DSC.

#### 2.3.3 Objective 3: unbiased brute-force exploration of the affine parameter space

The aim is to blindly assess a multitude of combinations from the full range of even remotely plausible values (see below) iteratively in a “brute force” approach. While not efficient, recent advances in computing power make such blind exploration of the whole parameter space increasingly feasible (Heule and Kullmann, 2017). This approach has the advantage of neither favoring one nor discouraging another solution, making it particularly suited for the processing of images that fall outside of the “normal” range (as implicitly defined by the starting estimates). Further, blind search approaches are insensitive to issues of bad initial starting estimates as the solutions are dynamically generated and are not vulnerable to local minima: as noted before, “the only method yielding global extreme solution is an exhaustive search” (Zitova and Flusser, 2003). Such “exhaustive search” approaches are usually avoided due to their vulnerability to the “combinatorial explosion” effects (Heule and Kullmann, 2017); this is particularly true as often, more elegant approaches are available (Schuster, 2000; Franz and Schölkopf, 2005; Gaur, 2012; Bapista and Poloczek, 2018). However, recent advances in computational power now severely reduce the time requirements, making such approaches practicable. Hence, in contrast to the “prior information” approach (using an empirically-derived set of starting estimates which is then iteratively refined; Ashburner et al., 1997), the approach suggested here is to first determine a very wide range of possible (not necessarily equally plausible) values and sample this parameter space systematically in a first step. From this first round, an “initial best guess combination” is selected, again based on the mutual information goodness-of-fit parameter (D’Agostino et al., 2004). This is then used as the basis for a second round with a finer-grained search window around the results from the first run. This “best final fit” is then supplied to SPM’s affine registration algorithm as the final, unbiased and optimized starting estimate, which is then iteratively refined as usual. As this objective requires a (p)redefined “normal range,” it involves several experiments.

##### 2.3.3.1 Experiment 3a

To assess the full range of normal in our large population from birth to old age, affine spatial normalization available within SPM12 was performed on all images, with regularization set to “none” (as the aim was to obtain unbiased solutions). All other settings were left at their respective default, including the standard tissue priors used in SPM12, spatial sampling, smoothing, and the iterative determination of whether the image origin or the center of the field of view provided better starting estimates. From the resulting affine matrices, the scaling in each dimension was derived, as well as the resulting overall scaling. These results provide the full range of scaling in each dimension.

##### 2.3.3.2 Experiment 3b

To cover this newly-defined normal range (cf. experiment 3a, above), the range of possible scaling parameters in each dimension was defined using *z*-scores. Due to the skewed distribution (higher scaling factors for minors in general, and young minors in particular, see below), the initial *z*-score range to-be-explored was chosen to be asymmetrical as well and was set from *z* = −5 to +15, for each dimension, amounting to 21 values each and a total of 9.261 unique initial scale factor combinations. To save processing time, the full parameter set of initial scaling parameters (*n* = 9.261) was by default systematically sampled to yield a subset of 1.000 combinations (see also experiment 3c, below), which were then applied to the input image. The initial best combination was again identified by the goodness-of-fit, and its scale factors were then modified in 2% steps from 90 to 110% and explored again (original position ±25 modifications in each direction = 51 scaling factors for each dimension = 153 final combinations) in a second round, intended to fine-tune the initial parameter set and to identify their best combination. This was then submitted as the starting estimate for the routine iterative optimization, and the resulting final fit from this was compared with the parameter obtained using the default approach, again using both image registration metrics.

##### 2.3.3.3 Experiment 3c

To ensure that the time-saving data reduction step does not impair image registration quality, all combinations (*n* = 9.261) instead of the reduced parameter set (*n* = 1.000) were explored, and results were compared.

##### 2.3.3.4 Experiment 3d

To assess the effect of the complete processing stream, the algorithm was evaluated in its entirety with all parameters set at defaults (use inhomogeneity correction and bias correction only if beneficial and use a reduced initial parameter set of *n* = 1,000).

### 2.4 Statistical analyses

Statistical testing was also performed within Matlab. Variables were initially tested regarding the assumption of normality using a Kolmogorov–Smirnov–Lilliefors-Test. If this assumption was met, two-sided Student’s *T*-tests were used, and mean values with standard deviations were reported. If the assumption of normality was not met, variables were compared using the non-parametrical Mann–Whitney-*U*-test, and median values with the median absolute deviation (MAD) were reported. For assessing the effect of an optimization step in a given group, paired *T*-Tests were used if applicable. For comparing proportions between groups, a chi-square test was used. Significance in all cases was assumed at *p* ≤ 0.05, Bonferroni-corrected for multiple comparisons where appropriate.

## 3 Results

### 3.1 Experiment 1a

**Figure 2:**
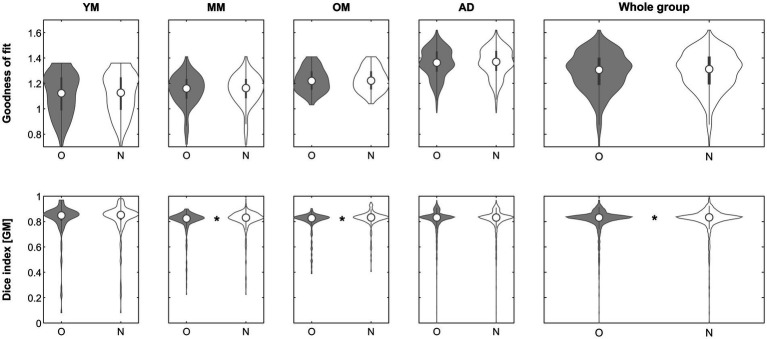
Violin plots of the effect of inhomogeneity removal (step 1) across groups on the goodness of fit (top row) and the gray matter Dice similarity index (bottom row). *Significant differences following correction for multiple comparisons. O, original; N, new approach. See text for details.

When assessing the impact of using the inhomogeneity-corrected image to perform affine registration (step 1), the goodness-of-fit criterion demonstrated superiority of the approach in the subgroups in 25/221 (YM; 11.3%), 55/324 (MM; 17.9%), 33/201 (OM; 16.4%), 338/1338 (AD; 25.2%) and in the whole group in 451/2081 (21.7%), respectively. The effect was significant in neither group. When assessing the impact on the Dice similarity index, the approach was beneficial in the subgroups in 17/221 (YM; 7.7%), 53/324 (MM; 16.4%), 33/201 (OM; 16.4%), 152/1338 (AD; 11.4%), and in 255/2081 (12.2%) in the whole group, respectively. The effect was significant in groups MM (*p* = 0.00347, Mann–Whitney), OM (*p* = 0.00224, Mann–Whitney) and in the whole group (*p* = 0.00347, Mann–Whitney) and remained so in all cases after correcting for five comparisons. See Figure 2 for details.

### 3.2 Experiment 1b

**Figure 3:**
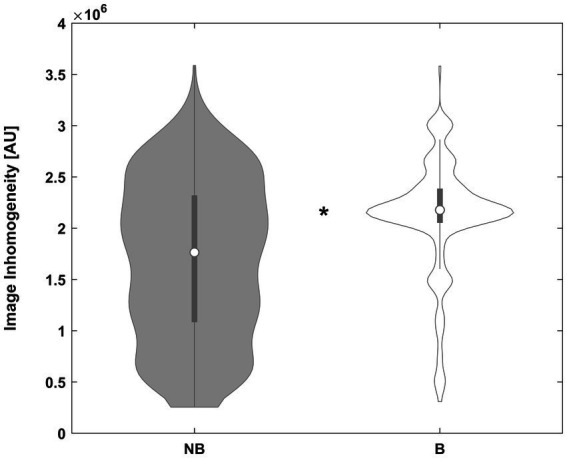
Violin plot of the inhomogeneity between subjects not benefitting from inhomogeneity correction (NB, left plot) and those benefitting from it (B, right plot), in arbitrary units (AU). *Significant difference. See text for details.

When comparing datasets where using the inhomogeneity-corrected image was beneficial with those where it was not, there was a clear effect of the amount of image inhomogeneity: significantly more inhomogeneity was present in those images where the approach was beneficial (889161.8 [MAD: 261223.3] versus 401044.7 [MAD: 226733.8] arbitrary units), resulting in *p* < 0.00001 (Mann–Whitney; see Figure 3). There was no independent effect of field strength (21.12% vs. 22.09% in 1.5T and 3.0T data, respectively, *p* > 0.05, chi-square).

### 3.3 Experiment 2

**Figure 4:**
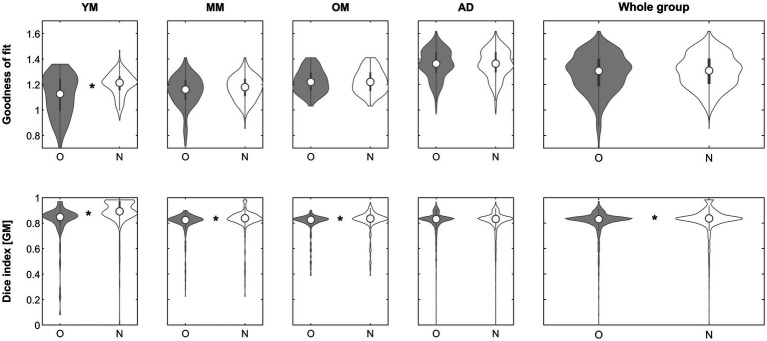
Violin plots of the effect of using a complexity-reduced approach (step 2) across groups on the goodness of fit (top row) and the gray matter Dice similarity index (bottom row). *Significant differences following correction for multiple comparisons. O, original; N, new approach. See text for details.

When assessing the impact of using the complexity-reduced image to perform affine registration (step 2), the goodness-of-fit criterion demonstrated superiority of the approach in the subgroups in 174/221 (YM; 89.6%), 97/324 (MM; 29.9%), 58/201 (OM; 28.9%), 687/1338 (AD; 51.3%) and in the whole group in 1016/2081 (48.8%), respectively. The effect was significant in group YM (*p* < 0.00001, Mann–Whitney) and remained so after correcting for five comparisons. When assessing the impact on the Dice similarity index, the approach was beneficial in the subgroups in 198/221 (YM; 89.6%), 301/324 (MM; 92.9%), 180/201 (OM; 89.6%), 769/1338 (AD; 57.5%) and in the whole group in 1448/2081 (69.6%), respectively. The effect was significant in groups YM (*p* < 0.00001, Mann–Whitney), MM (*p* < 0.00001, Mann–Whitney), OM (*p* = 0.00018, Mann–Whitney) and in the whole group (*p* = 0. 00001, Mann–Whitney) and remained so in all cases after correcting for five comparisons. See Figure 4 for details.

### 3.4 Experiment 3a

**Figure 5:**
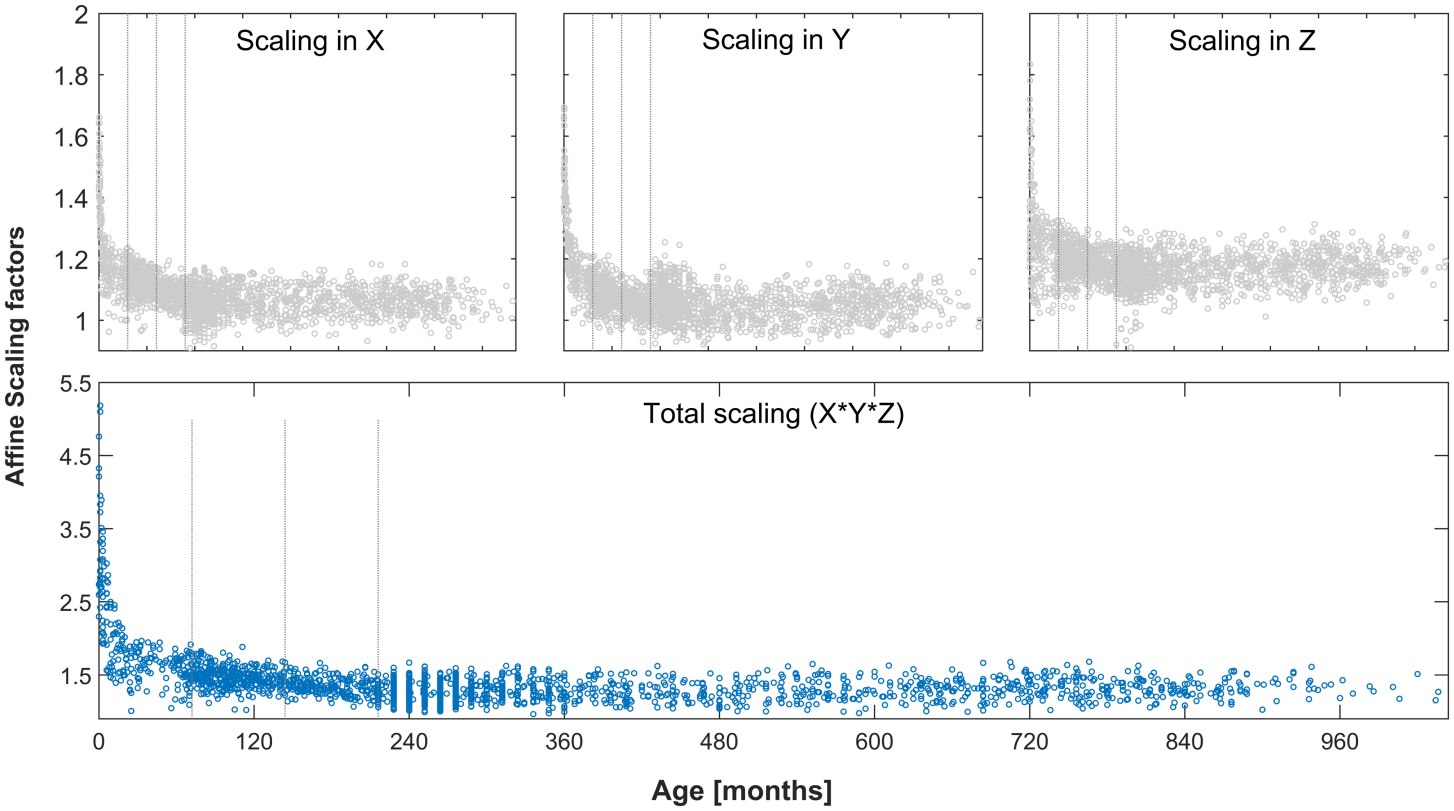
Scaling factors in each dimension (gray markers, top plots) and total scaling factor (blue markers, bottom plot) as a function of age, derived from the whole cohort. Gray lines indicate groups (young, middle, and old minors). All differences in total scaling between minors and adults were significant after correcting for multiple comparisons. See text for details.

When visually assessing the scaling factors, a clear effect of age was present and most pronounced in the youngest participants. Confirming this, total scaling was significantly different between each group of minors and adults (YM vs. AD, MM vs. AD, and OM vs. AD; each *p* < 0.001, Mann–Whitney-U) and remained significant after correcting for three comparisons. See Figure 5 for details. The parameters describing the scaling across the whole age range in each dimension are also provided in Table 2.

**Table 2:** Empirically-derived scaling factors across the whole age range (experiment 3a): mean and standard deviation, as well as actually-observed minimum and maximum values.

	Mean	*SD*	Minimum [actual value]	Maximum [actual value]	Minimum [*z* = −5]	Maximum [*z* = 15]	“MNI”
*X*-dimension	1.088	0.078	0.909	1.661	0.698	2.258	1.069
*Y*-dimension	1.075	0.086	0.895	1.694	0.645	2.365	1.0339
*Z*-dimension	1.179	0.073	0.911	1.834	0.814	2.274	1.113
Total scaling	1.401	0.335	0.965	5.184			1.2301

See Figure 4. Also provided are the minimum and maximum values (as defined by *z*-scores) here explored by the algorithm. For comparison, the scaling applied by default within SPM12 using the “MNI” regularization scheme is also provided.

### 3.5 Experiment 3b

**Figure 6:**
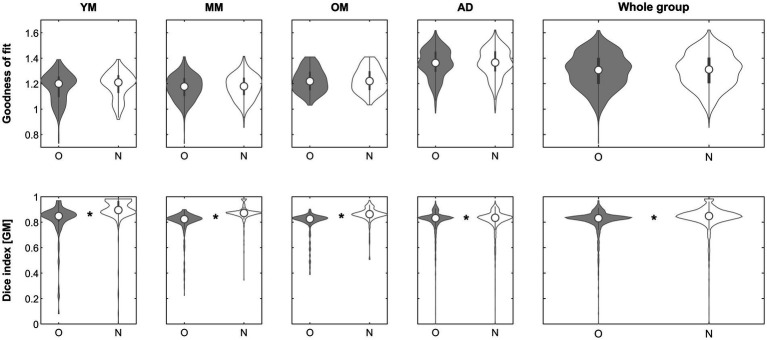
Violin plots of the effect of using an iterative brute-force exploration (step 3) across groups on the goodness of fit (top row) and the gray matter Dice similarity index (bottom row). *Significant differences following correction for multiple comparisons. O, original; N, new approach. See text for details.

When assessing the impact of using iterative processing only to perform affine registration (step 3), the goodness-of-fit criterion demonstrated superiority of the approach in the subgroups in 197/221 (YM; 89.1%), 306/324 (MM; 94.4%), 191/201 (OM; 95%), 1261/1338 (AD; 94.2%) and in the whole group in 1955/2081 (93.9%), respectively. The effect was significant in neither group. When assessing the impact on the Dice similarity index, the approach was beneficial in the subgroups in 193/221 (YM; 87.3%), 301/324 (MM; 92.9%), 180/201 (OM; 89.6%), 761/1338 (AD; 56.9%) and in the whole group in 1435/2081 (69%), respectively. The effect was significant in groups YM (*p* < 0.00001, Mann–Whitney), MM (*p* < 0.00001, Mann–Whitney), OM (*p* < 0.00001, Mann–Whitney), AD (*p* = 0.00687, Mann–Whitney) and in the whole group (*p* < 0.00001, Mann–Whitney) and remained so in all cases after correcting for five comparisons. See Figure 6 for details.

### 3.6 Experiment 3c

When assessing the impact of using a reduced initial parameter set for iterative processing (*n* = 1.000 vs. *n* = 9.621), the mutual information “goodness of fit” criterion demonstrated no superiority of the extensive approach in either the whole group or any of the subgroups (all *p* > 0.05, Mann–Whitney [3] and 2-sample *t*-test [2]). Similarly, the Dice similarity index was not significantly different between the extensive and the reduced approach in either the whole group or any of the subgroups (all *p* > 0.05, Mann–Whitney).

### 3.7 Experiment 3d

**Figure 7:**
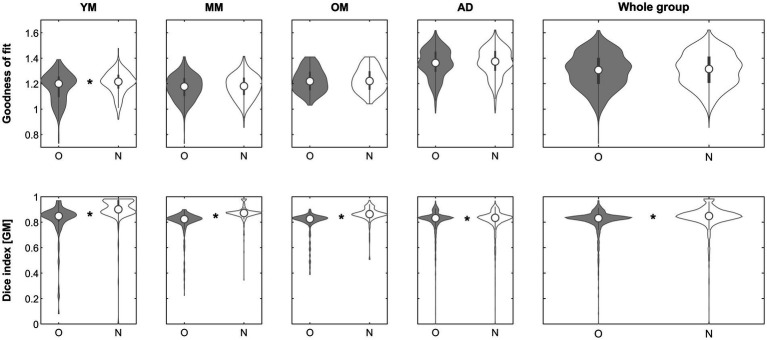
Violin plots of the effect of using the complete processing stream (steps 1–3) across groups on the goodness of fit (top row) and the gray matter Dice similarity index (bottom row). *Significant differences following correction for multiple comparisons. O, original; N, new approach. See text for details.

When assessing the impact of using the complete processing stream (steps 1, 2, and 3 with default settings, i.e., inhomogeneity correction [when beneficial], complexity-reduced processing [when beneficial] and iterative processing [with initial *n* = 1,000]) to perform affine registration, the goodness-of-fit criterion demonstrated superiority of the approach in the subgroups in 210/221 (YM; 95%), 312/324 (MM; 96.3%), 195/201 (OM; 97%), 1293/1338 (AD; 96.6%) and in the whole group in 2010/2081 (96.6%), respectively. The effect was significant in group YM (*p* = 0.00496, Mann–Whitney) and in the whole group (*p =* 0.04118, Mann–Whitney) but survived comparison for 5 multiple comparisons only in group YM. When assessing the impact on the Dice similarity index, the approach was beneficial in the subgroups in 198/221 (YM; 89.6%), 298/324 (MM; 92%), 180/201 (OM; 89.6%), 753/1338 (AD; 56.3%) and in the whole group in 1429/2081 (68.7%), respectively. The effect was significant in groups YM (*p* < 0.00001, Mann–Whitney), MM (*p* < 0.00001, Mann–Whitney), OM (*p* < 0.00001, Mann–Whitney), AD (*p* = 0.00643, Mann–Whitney) and in the whole group (*p* < 0.00001, Mann–Whitney) and remained so in all cases after correcting for five comparisons. See Figure 7 for details.

## 4 Discussion

This technical note was meant to address three main objectives in a large population across a very wide age range: one, can affine spatial normalization be improved by performing an initial bias correction; two, can affine spatial normalization be improved by initially using a complexity-reduced input, and three, can affine spatial normalization be improved by performing an unbiased, brute-force exploration of numerous affine scaling parameter combinations (see Figure 1 for an overview). Two metrics were used, the goodness of fit (the parameter used internally to optimize affine processing) and Dices similarity index (reflecting the actual impact the procedure has on the resulting segmented maps). Results shall now be discussed in succession.

With regard to objective 1, does affine processing benefit from prior inhomogeneity removal, the approach was beneficial in only about 22% of images. However, it was beneficial particularly in those where more image inhomogeneity was present. While the goodness of fit was not significantly better in either group, the resulting segmentation partitions benefitted significantly in the whole and in two of the minor groups (Figure 2), incidentally confirming the complementary nature of our two indicators. These results demonstrate that image inhomogeneity indeed interferes with image registration and confirms previous suggestions to this effect (Vovk et al., 2007; Wilke et al., 2017; SPM Manual, 2023). While it may seem surprising that there was no independent effect of field strength, it must be borne in mind that many of the images from higher-field datasets were acquired more recently (such as the CMIND or the IXI datasets). Hence, there may be an effect of particular scanners, improved image acquisition, or better shimming techniques that could outweigh a possible effect of field strength *per se*. While the approach was only beneficial in a minority of our datasets, our results suggest that it may be particularly helpful in datasets where inhomogeneity is prominent (Figure 3), such as images from ultra-high-field strength scanners (Kraff and Quick, 2017). In the algorithm, it is therefore suggested by default but will only be used if found to improve the result.

With regard to objective 2, using a complexity-reduced input image for the initial matching, the approach was beneficial in about 49% of images (Figure 4). While it resulted in a significantly better fit only in the young group, the impact on the resulting gray matter partitions was significant in all minor as well as the whole group, clearly suggesting an effect of age. This demonstrates that the brains of infants and children in particular seem to be matched better if their complexity is reduced. Reducing the complexity of the input image likely reduces the complexity of the differences between the individual brain and the template, which, being a group average, is complexity-reduced by default; consequently, it must be expected to be of the strongest benefit where these differences are large. This effect confirms that scaling differences alone (see below) are not the only hindrance when it comes to matching these “unusual” brains to an adult template, in line with previous observations (Sanchez et al., 2012; Wilke et al., 2017; Dong et al., 2020). It must be borne in mind that all images in this dataset were already “nicely oriented” as suggested (Ashburner, 2021; Larroque, 2021), but this image position, too, is iteratively optimized as the positional information from each step is automatically used as the basis for the next step if it provides a better fit than the original one. This approach may consequently be more beneficial in images with a less-than-optimal initial orientation. Further, matching using the whole head may seem rather course, and tissue-specific matching would theoretically be advantageous. However, increasing accuracy is achieved in the later stages of unified segmentation, where the initial affine parameters (provided here) are iteratively updated and optimized. Therefore, prior skull stripping is not part of the segmentation algorithm of SPM12 (Wang et al., 2018) as non-brain tissue is explicitly modelled during this process. The aim of the approach presented here was to optimize the initial starting estimates to provide a better basis for this later optimization; hence, the balance between greater robustness and greater accuracy can be weighed toward the former in this particular instance. An option to base the complexity-reduced matching on brain tissue only (Wilke et al., 2011) was also implemented and tested; however, this approach did not show superiority over the head-based approach and requires an additional processing step; it is therefore disabled by default. Of note, the complexity-reduced approach is implemented in the algorithm such that, if no 2-class prior matching the supplied 6-class prior is found, one is generated “on the fly” (such that it can seamlessly be used with each supplied prior, standard or custom). As with the inhomogeneity correction, it is suggested by default but, if the goodness-of-fit parameter is not improved, its solution will not be used.

With regard to objective 3, several experiments were performed, to first define and then blindly explore the full range of even remotely plausible values. With regard to experiment 3a, our results show that a very wide range of values must be expected in a healthy human population, with a clear and substantial effect of age on the total scaling factor especially in younger children (Figure 5). This underlines the fact that appropriate data processing for the brains of children in general, and younger children in particular, requires solutions that differ from standard adult reference data, as suggested before (Wilke et al., 2008; Sanchez et al., 2012; Bednarz and Kana, 2018). In the context of affine transformation, the issue arises when starting estimates from adults only is used, as in SPM12 (initially generated in Ashburner et al., 1997 and subsequently updated). The effect is most pronounced in the Z-dimension (inferior–superior), where the actually observed minimum and maximum values differ by a factor of more than 2. Clearly, the higher mean scaling factors in our population along each dimension (when compared to the current standard from and for adults, cf. Table 2) are due to the inclusion of minors. This is confirmed by assessing the scaling factors from our adult group only, which are 1.0587 ± 0.0443, 1.0513 ± 0.053, and 1.161 ± 0.0514 (mean ± SD), for scaling in *X*, *Y*, and *Z*, respectively. These values are much closer to the default values used in SPM12 (cf. Table 2), confirming that those clearly are appropriate for use with datasets conforming to the assumptions (i.e., adults).

With regard to experiment 3b, does an unbiased application of a wide range of affine scaling parameter combinations alone result in an improved fit, the initial answer is no. While the approach is clearly beneficial and improves the goodness of fit in the vast majority of datasets, independently of group (range, 89–95% of subjects), the differences do not reach significance. Interestingly, however, segmentation quality improves significantly in all groups, minors and adults alike (Figure 6). Experiment 3c demonstrates that results do not suffer from using a less-extensive exploration of the full parameter space (*n* = 1.000 vs. *n* = 9.621), which is reassuring and helpful in practical terms as this reduction naturally reduces processing time substantially. Finally, experiment 3d demonstrates that the full processing stream (using steps 1, 2, and 3 with their respective defaults), the goodness of fit improves in 95–97% of images, although the effect is significant only in the young minors group. The segmentation partitions, however, benefit significantly across all groups (Figure 7), but substantially more so in the minor groups (89.6/92/89.6%, respectively) than in adults (56.3%). This demonstrates that the strength of the approach must be expected to come to bear mainly for “unusual” datasets where the *a priori* assumptions may be off. In the algorithm, the goodness of fit will be compared to the “traditional” approach, and will not be used if no improvement is found. Hence, the approach will only result in an equal-or-higher goodness of fit, as in the minority of images not benefitting from the approach, the traditional approach will be used. By default, information about the two solutions and the “better choice” is visually presented and saved as a graphics file, allowing for later quality control. Some examples from each group are shown in Supplementary Figure S1.

In *post hoc* analyses, our results also illustrate that the mutual information goodness-of-fit criterion itself shows a clear age-dependency, irrespective of the approach, when using an adult template. Here, the fit achieved in all minors (groups YM, MM, and OM) is significantly lower than that in adults (cf. Figure 7; each *p* < 0.001, Mann–Whitney-*U*, all surviving Bonferroni-correction for 3 comparisons). The same is true for the Dice coefficient (YM vs. AD, *p* < 0.001; MM vs. AD, *p* = 0.0022; OM vs. AD, *p* = 0.00656, all Mann–Whitney-*U*, all surviving Bonferroni-correction for 3 comparisons). This demonstrates that the brains of minors (all groups) are not equally-well fitted to the standard adult template as are those of adults, which again is generally in line with previous observations (Sanchez et al., 2012; Richards et al., 2016; Wilke et al., 2017). However, it is surprising that this effect is already clear, and significant, for the affine spatial normalization, where previously, an age-dependency of the scaling parameters was not found after the age of about 6 years (Muzik et al., 2000; Wilke et al., 2002). The here-used goodness-of-fit parameter and the Dice coefficient, therefore, seem to be more sensitive to the differences between a child’s brain and the adult template. To test this hypothesis, each group of minors was matched to an age-appropriate template (Wilke et al., 2017). Here, the goodness of fit was significantly higher when matching to a custom template than when matching to an adult template for all groups of minors (each *p* < 0.001, Mann–Whitney-*U*, Bonferroni-corrected for 3 comparisons). This was also the case for the Dice coefficient for groups YM and MM (each *p* < 0.001, Mann–Whitney-*U*, Bonferroni-corrected for 3 comparisons), but not for group OM (*p =* 0.0357, Mann–Whitney-*U*, not surviving Bonferroni-correction). This underlines the positive effect of using an age-appropriate template even for the affine registration, in younger subjects in particular.

### 4.1 Implementation and resulting “pipeline”

The algorithm was implemented by modifying a single file (function spm_maff8 from the latest SPM12 developmental version, last modified August 17, 2023). It will be executed automatically if spatial normalization is performed in the context of unified segmentation (Ashburner and Friston, 2005), but not otherwise (e.g., if called from the command line or from other software solutions), unless specifically requested. With the default settings, inhomogeneity correction will be performed first, and solutions from the original as well as the bias-corrected image are generated. The solution with the better fit will then be used to inform the next step (complexity-reduced processing), from which (again) the better of the two solutions will be used to inform the last step (iterative processing). Yet again, the solution obtained here will be compared to the standard approach, and the one leading to a better fit is used as the final solution. As already mentioned, this process ensures that no solution will be worse than the solution obtained by the standard, original approach.

### 4.2 Possible limitations of this study

It was argued that using brute force to solve a problem is unsatisfactory as “brute force does not contribute to understanding the problem” (Heule and Kullmann, 2017). However, it has the undeniable advantage of being blind, and hence, unbiased. A more elegant approach might be to reparameterise the results obtained in the different age groups to instead be modelled by age, sex, and/or field strength, as done with structural MRI data in the past (Wilke et al., 2008, 2017). This would provide a theoretically “optimal” solution for the affine parameter starting estimates, based on technical and/or the individual subject’s demographics characteristics. However, the range of normal even among subjects of similar age is quite large, as can be seen from Figure 5. Hence, the theoretically “optimal” solution for a given subject may still not be optimal at all for an individual at hand, and be less-suited than a solution found blindly. Further, this would introduce new assumptions which may or may not apply (e.g., in cases of macro- or microcephaly), and would be less blind to “non-modelled anomalies” such as brain lesions. Also, the interdependency, and potentially, multicollinearity (Alin, 2010) of the three input variables (changes in *X* and *Y* might influence *Z*, and vice versa) would make any such optimization complex. Additionally considering that any optimization approach would also require “non-trivial computation time” to determine, or exclude, the next solution (McLeod et al., 2018), this does not seem sensible considering that each single computation of the goodness-of-fit criterion used here only takes a few milliseconds when using parallel processing. Therefore, while admittedly not an elegant approach, the 2–3 min of extra processing time (usually less time than required for the ensuing standard segmentation itself) per subject to find the best-possible, individual parameter set blindly seems to be time well-invested.

It could also be argued that the inhomogeneity removal should be done using one of the many other approaches available out there (Sled et al., 1998; Vovk et al., 2007; Weiskopf et al., 2011; Ganzetti et al., 2016), particularly if additional MRI data was obtained that allows to explicitly model, and remove, such inhomogeneities (Tabelow et al., 2019). However, while this is of course technically feasible, mixing different data processing approaches within one processing stream is not always a good idea, due to the unpredictable interactions between them. Using “unified segmentation” to remove inhomogeneity also has the practical advantage of not requiring (interactions with) other software solutions, but of course such solutions could be implemented.

Our sample was, by design (multiple sites, scanners, and sequences) inhomogeneous, including some images acquired a long time ago. Apart from image inhomogeneity, no attempt was made to identify factors that may have contributed to some images benefitting more from the approach presented here. However, the overall clear benefit of the individual steps as well as the combined approach in a diverse sample such as this one clearly demonstrates its real-world usability.

Our approach “only” explored combinations of the three scaling parameters in each dimension, as these are also those for which starting estimates are used (Ashburner et al., 1997). It would be theoretically more appealing to blindly explore each of the 12 affine parameters, but this would result in not 9.261 combinations (21^3^) but in 7.35583 × 10^15^ (21^12^) which is not feasible. Also, the “other” parameters encoding shears, rotations, and shifts, are already determined by an early, almost rigid-body matching using either the center of the image or the origin. This initial match is then optimized further using each newly-generated affine matrix. It therefore does not seem necessary to also iterate over all of these, clearly less-important parameters.

Finally, the *z*-score range that was explored in this study was deliberately chosen to be extremely wide and consequently also includes unrealistically small or large values; to cover the range of values actually observed in this sample, a range of *z* = −4:9 would have been sufficient (instead of the −5:15 range actually chosen; cf. Table 2). This means that, in most cases, a wider range than realistically necessary is explored, which is not very efficient. Consequently, this setting can be adapted in the code to restrict the initially-explored parameter space. Ultimately, however, the setting that is more relevant to how long the code actually runs is how fine-grained that space is finally explored as this is the speed-limiting step (default: 1.000 values, cf. experiment 3c).

## 5 Summary and conclusion

A lower vulnerability toward image inhomogeneity and “unusual brains” of the popular affine transformation step in SPM12 could be achieved using an initial bias correction step and using a complexity-reduced input image, respectively. Following the exploration of a wider range of normal values, it was also shown that the vast majority of datasets benefit from applying a blind, brute-force search of all possible parameter combinations. At each step, results are compared with the traditional approach to ensure that no solution worse than the original one is used, such that effectively, only an equal or better solution is obtained. For the complete processing stream, significant superiority is demonstrated across the whole group, as well as in young minors. The approach, while more time-consuming, may therefore be particularly beneficial when exploring inhomogeneous and/or unusual datasets, such as those stemming from high-field scanners and those containing data from children in general, and from younger children in particular.

## Data availability statement

The original contributions presented in the study are included in the article/Supplementary material, further inquiries can be directed to the corresponding author. The code to implement the approach described here will be made available for free download on the Experimental Pediatric Neuroimaging group’s website at: https://www.medizin.uni-tuebingen.de/en-de/das-klinikum/einrichtungen/kliniken/kinderklinik/forschung/forschung-iii/software# (short link: https://tinyurl.com/EPN-software).

## Ethics statement

The data used was obtained from public repositories. Ethical approval or informed consent was therefore not required for this study in accordance with the local legislation and institutional requirements.

## Author contributions

MW: Conceptualization, Data curation, Formal analysis, Funding acquisition, Investigation, Methodology, Project administration, Resources, Software, Supervision, Validation, Visualization, Writing – original draft, Writing – review & editing.

## Supplementary material

SUPPLEMENTARY FIGURE S1 Examples of actual algorithm output for the three subjects with the biggest improvement of the mutual information criterion per group (Y = young; M = middle, O = old children; A = adults). Note more realistic scaling (e.g., case Y1 or case M3) or improved rotation of the input data (e.g., case Y3).
