# Supplementary material for: A three-step, “brute-force” approach toward optimized affine spatial normalization
Source: Front Comput Neurosci. 2024 Jul 8;18:1367148. doi: 10.3389/fncom.2024.1367148 (PMC11260722; doi:10.3389/fncom.2024.1367148)
Supplement: Supplementary file 2 [file Data_Sheet_1.docx]

**A three-step, “brute-force” approach towards
optimized affine spatial normalization**

Marko Wilke ^1,2^

*^1^* *Department of Pediatric Neurology and Developmental Medicine, Children’s Hospital, and*

*^2^ Experimental Pediatric Neuroimaging, Children’s Hospital and Department of Neuroradiology,
University of Tübingen, Germany*

**Correspondence**:

Marko.Wilke@med.uni-tuebingen.de

**Supplemental Material**

**Supplementary material: detailed listing of subject IDs from all datasets**

**Dataset 1**: From the NIH Study of normal brain development (NIH, Bethesda, MD, USA), URL: https://www.bic.mni.mcgill.ca/nihpd_info/, 500 subjects with the following IDs were used: 1001, 1002, 1003, 1004, 1005, 1007, 1008, 1009, 1010, 1011, 1012, 1013, 1014, 1015, 1016, 1017, 1018, 1019, 1020, 1021, 1023, 1024, 1025, 1026, 1027, 1028, 1029, 1030, 1031, 1032, 1033, 1034, 1035, 1036, 1037, 1038, 1039, 1040, 1041, 1042, 1043, 1044, 1045, 1046, 1047, 1051, 1052, 1053, 1054, 1055, 1056, 1057, 1058, 1059, 1061, 1062, 1063, 1064, 1065, 1066, 1067, 1068, 1069, 1070, 1071, 1072, 1073, 1074, 1075, 1076, 1077, 1078, 1079, 1080, 1081, 1082, 1083, 1084, 1085, 1086, 1087, 1088, 1089, 1090, 1091, 1092, 1093, 1094, 1095, 1096, 1097, 1098, 1099, 1100, 1102, 1103, 1104, 1105, 1106, 1107, 1108, 1109, 1110, 1111, 1112, 1113, 1114, 1115, 1116, 1117, 1118, 1119, 1121, 1122, 1123, 1124, 1125, 1126, 1127, 1128, 1129, 1130, 1131, 1132, 1133, 1134, 1136, 1137, 1138, 1139, 1140, 1141, 1142, 1143, 1144, 1145, 1146, 1147, 1148, 1149, 1150, 1151, 1153, 1154, 1155, 1157, 1158, 1159, 1160, 1161, 1162, 1163, 1164, 1165, 1166, 1167, 1168, 1169, 1170, 1171, 1172, 1173, 1174, 1175, 1176, 1177, 1178, 1179, 1180, 1181, 1182, 1183, 1184, 1185, 1186, 1187, 1188, 1189, 1190, 1191, 1192, 1193, 1194, 1195, 1196, 1197, 1199, 1200, 1201, 1202, 1203, 1204, 1205, 1206, 1207, 1208, 1209, 1210, 1211, 1213, 1215, 1216, 1217, 1218, 1219, 1220, 1221, 1222, 1223, 1224, 1225, 1226, 1227, 1228, 1229, 1230, 1231, 1232, 1233, 1234, 1235, 1236, 1237, 1238, 1239, 1240, 1241, 1242, 1243, 1244, 1245, 1246, 1247, 1248, 1249, 1250, 1251, 1252, 1253, 1254, 1255, 1257, 1258, 1259, 1260, 1262, 1263, 1264, 1265, 1266, 1267, 1268, 1269, 1270, 1271, 1272, 1273, 1274, 1275, 1276, 1277, 1278, 1279, 1280, 1281, 1282, 1283, 1284, 1285, 1286, 1287, 1288, 1289, 1290, 1291, 1292, 1294, 1295, 1297, 1298, 1299, 1300, 1301, 1302, 1303, 1304, 1305, 1306, 1307, 1308, 1309, 1310, 1311, 1312, 1313, 1314, 1315, 1316, 1317, 1318, 1319, 1320, 1321, 1322, 1323, 1324, 1325, 1326, 1328, 1329, 1330, 1331, 1332, 1333, 1334, 1335, 1336, 1337, 1338, 1339, 1340, 1341, 1342, 1343, 1344, 1346, 1348, 1349, 1350, 1351, 1352, 1353, 1354, 1355, 1356, 1357, 1358, 1359, 1360, 1361, 1362, 1363, 1364, 1365, 1366, 1367, 1368, 1369, 1370, 1371, 1372, 1373, 1374, 1375, 1376, 1377, 1378, 1379, 1380, 1381, 1382, 1383, 1384, 1385, 1386, 1387, 1388, 1389, 1390, 1391, 1392, 1393, 1394, 1395, 1396, 1397, 1398, 1399, 1400, 1401, 1402, 1403, 1404, 1405, 1406, 1407, 1408, 1409, 1410, 1411, 1413, 1414, 1415, 1416, 1417, 1418, 1419, 1420, 1421, 1422, 1423, 1424, 1425, 1426, 1427, 1428, 1429, 1430, 1431, 1432, 1433, 1435, 1436, 1437, 1438, 1439, 1441, 1443, 1444, 1445, 1446, 1447, 1448, 1450, 1451, 1452, 1453, 1454, 1455, 1457, 1458, 1459, 1462, 1463, 1464, 1465, 1466, 1469, 1470, 1471, 1472, 1473, 1475, 1476, 1477, 1478, 1479, 1480, 1481, 1482, 1483, 1484, 1485, 1486, 1487, 1488, 1489, 1490, 1491, 1494, 1495, 1496, 1498, 1499, 1500, 1501, 1502, 1503, 1504, 1505, 1506, 1507, 1508, 1510, 1511, 1513, 1514, 1515, 1516, 1517, 1518, 1519, 1521, 1522, 1525, 1528, 1531, 1532, 1533, 1534, 1538, 1542, 1543, 1546, 1547, 1550, 1551, 1552, 1553, 1555.

Please note: this manuscript reflects the views of the author and may not reflect the opinions or views of the Brain Development Cooperative Group Investigators or the NIH. The contract numbers for the NIH MRI study of normal brain development were N01-HD02-3343, N01-MH9-0002, and N01-NS-9-2314, 2315, 2316, 2317, 2319 and 2320. A listing of the participating sites and a complete listing of the study investigators can be found on p6 of the study protocol available at the homepage provided above.

**Dataset 2**: From the Cincinnati MR Imaging of NeuroDevelopment (C-MIND) study, URL: https://research.cchmc.org/c-mind/, 235 subjects with the following IDs were used: 00F011_C, 00F012_C, 00M002_U, 00F001_C, 00F015_C, 00M003_C, 00M016_C, 00M018_C, 00M042_C, 00M065_C, 00F037_C, 00F020_C, 00M002_C, 00M048_C, 00F007_C, 00F036_C, 00M050_C, 00M055_C, 00M034_C, 01F011_C, 01M001_C, 01F043_C, 01F032_C, 01F033_C, 01F038_C, 01F020_C, 01M005_C, 01F037_C, 01M035_C, 01F015_C, 01M029_C, 01F014_C, 01F036_C, 01M014_C, 01M002_U, 01M008_C, 02F003_C, 01F044_C, 02F023_C, 02M005_U, 02F031_C, 02M034_C, 02F020_C, 02F029_C, 02M022_C, 02M015_C, 02F011_C, 02F043_C, 02M018_C, 02F028_C, 02F021_C, 02F022_C, 02F044_C, 02F035_C, 02F016_C, 03M033_C, 03F011_C, 03M035_C, 02M021_C, 03F025_C, 03M005_C, 03M017_C, 03F033_C, 03F026_C, 03M011_C, 03M034_C, 03F034_C, 03F001_C, 03F003_C, 03F017_C, 03F023_C, 04F022_C, 03M001_C, 03M019_C, 04F019_C, 04M005_C, 04F001_C, 04F007_C, 04M002_C, 04F018_C, 05F017_C, 05M004_C, 05F003_C, 05F016_C, 05M003_C, 05F015_C, 05M002_C, 05F006_C, 05M001_C, 05F002_C, 05F012_C, 06F014_C, 06F010_C, 06M018_C, 06M011_C, 06F003_C, 06F004_C, 06F016_C, 06M007_C, 06F015_C, 06M014_C, 06M002_C, 06M008_C, 07F004_C, 07M005_C, 07F001_C, 07F012_C, 07M007_U, 07F003_U, 07M008_U, 07M006_U, 07F002_U, 07M014_C, 07M009_C, 07F010_C, 07M002_U, 07F002_C, 07M017_C, 07F003_C, 08F020_C, 07M005_U, 07M008_C, 08F013_C, 07M010_C, 08M008_C, 07M003_C, 08M002_U, 08F004_C, 08F010_U, 08F016_C, 08M011_C, 08F007_U, 08F005_U, 08F010_C, 08M002_C, 08M012_C, 08F005_C, 08M003_U, 09M005_C, 08F003_C, 08F006_C, 08F008_C, 08F014_C, 08F019_C, 08F006_U, 08F009_U, 08F017_C, 08F021_C, 08M004_U, 08M010_C, 08M017_C, 08F008_U, 08F015_C, 08F002_U, 09F002_C, 09F004_U, 09M003_U, 09M007_C, 09M004_C, 09F010_U, 09F002_U, 09F006_U, 09M004_U, 09M014_C, 09M021_C, 09F017_C, 09M002_U, 09M005_U, 09F005_U, 09F009_C, 09M013_C, 09F007_U, 09F009_U, 09M001_U, 09F012_C, 10F002_C, 10F005_C, 10F001_C, 10M002_C, 10F006_C, 10M004_C, 11F007_C, 11M002_C, 11M005_C, 11M001_C, 11F004_C, 11F008_C, 11M007_C, 12M002_C, 12F007_C, 12M008_C, 12F001_C, 12M007_C, 12F004_C, 12F005_C, 12M003_C, 13F005_C, 13F003_C, 13F004_C, 13F007_C, 13M003_C, 13M018_C, 13M015_C, 13M001_C, 13M004_C, 14F006_C, 14F003_C, 14F002_C, 14F001_C, 14M012_C, 14M006_C, 14F009_C, 14M014_C, 15M022_C, 15M015_C, 15F001_C, 15F002_C, 15M013_C, 16F007_C, 15M005_C, 16M010_C, 16F005_C, 16M002_C, 16M007_C, 16M014_C, 17F002_C, 17M004_C, 17M010_C, 17F004_C, 17M003_C, 18M001_C, 18F004_C, 18F011_C, 18F002_C, 18F012_C.

Please note: data presented in this work was obtained from the database known as Cincinnati MR Imaging of NeuroDevelopment (C-MIND), provided by the Pediatric Functional Neuroimaging Research Network at https://research.cchmc.org/c-mind/. This Network and the resulting C-MIND database was supported by contract from the Eunice Kennedy Shriver National Institute of Child Health and Human Development (HHSN275200900018C).

**Dataset 3:** From *The 1000 Functional Connectomes Project*, URL: <http://www.nitrc.org/ir/app/action/ProjectDownloadAction/project/fcon_1000>, 783 subjects with the following IDs were used: from Ann Arbor: 306, 4111, 4619, 5580, 7921, 11043, 13636, 13959, 15846, 16960, 18546, 18698, 20317, 26099, 28433, 30250, 30421, 33437, 34781, 38614, 39635, 39923, 42616, 43409, 45569, 45660, 46727, 47191, 47659, 49687, 50859, 51248, 53269, 53959, 56028, 56686, 57025, 57196, 59573, 62819, 64831, 64969, 66506, 70106, 72215, 72416, 73168, 73812, 75922, 78151, 82334, 85257, 86367, 87745, 90127, 90950, 96621, 97518, 98007, 99692. From Atlanta: 354, 368, 6870, 7145, 15817, 18219, 18702, 24972, 26938, 32093, 49816, 53122, 55652, 58250, 59806, 60499, 61442, 61902, 71337, 72096, 72971, 75153, 76280, 81596, 86323, 91049. From Baltimore: 17017, 19738, 23750, 23927, 29158, 30072, 31837, 37548, 52358, 54257, 54329, 73823, 76160, 77572, 80221, 81887, 85922, 86414, 90658, 90893, 91622, 94042, 96234. From Bangor: 31, 1903, 3557, 4097, 14388, 27519, 36736, 46870, 48632, 61418, 61908, 63767, 66585, 68050, 73082, 77520, 81464, 82625, 87568, 91556. From Beijing: 440, 1018, 1244, 2403, 4050, 4191, 5267, 6880, 6899, 7144, 7716, 7717, 8001, 8251, 8455, 8816, 8992, 10186, 10277, 10869, 10973, 11072, 11344, 12220, 14238, 15441, 16091, 16943, 17093, 17159, 17315, 17586, 17603, 17642, 17688, 18326, 18758, 18960, 19642, 19974, 20127, 20246, 20765, 20948, 21115, 22201, 22595, 22661, 22715, 22890, 26713, 28206, 28403, 28698, 28792, 28801, 28907, 28965, 29590, 29785, 30272, 30310, 30556, 30616, 30988, 31058, 31729, 32517, 32587, 33747, 33943, 33991, 34895, 34943, 35309, 35776, 35806, 36580, 36942, 37602, 38602, 39725, 40037, 40427, 41170, 41621, 42512, 42555, 42843, 43290, 44573, 45552, 46058, 46259, 46541, 48501, 48563, 48676, 49782, 50498, 50873, 50972, 50985, 51015, 51586, 52044, 52259, 53572, 53998, 54890, 55301, 55541, 55736, 55856, 56136, 56659, 56703, 56757, 58029, 58332, 58614, 59347, 59448, 61961, 62083, 62438, 62843, 62966, 64923, 65467, 65659, 66158, 66528, 66781, 66889, 67435, 67844, 68012, 68597, 69518, 69696, 71693, 72654, 72678, 73098, 73245, 73279, 73421, 74386, 74587, 75878, 76377, 77440, 80163, 80551, 80569, 80927, 81062, 81074, 82352, 82426, 82714, 82826, 82980, 83430, 83624, 83728, 85030, 85543, 85818, 86114, 87089, 87776, 88306, 88947, 89088, 89238, 89592, 89742, 89941, 91145, 91399, 91952, 92430, 92490, 92544, 92602, 92799, 92859, 93689, 93856, 94536, 95575, 95755, 96163, 97442, 98353, 98617. From Berlin: 6204, 6716, 12855, 18913, 23506, 27536, 27711, 27797, 28092, 33248, 38279, 40143, 47066, 47791, 49134, 54976, 57028, 67166, 75506, 77281, 85681, 86111, 91116, 91966, 95068, 97162. From Cambridge: 156, 294, 1361, 2591, 2953, 4187, 4270, 4491, 4665, 5306, 5453, 6037, 6272, 6987, 7413, 7798, 7902, 8204, 8588, 8723, 8947, 9015, 9397, 9633, 10268, 10619, 11388, 12346, 13093, 13187, 13216, 13902, 14183, 14194, 14278, 15172, 15258, 15432, 15905, 16122, 16390, 16846, 17584, 17737, 17772, 18295, 18449, 19717, 20389, 20543, 20563, 21755, 23780, 23869, 24670, 24757, 25044, 25058, 25099, 26348, 27065, 27230, 27613, 27796, 29044, 29425, 29800, 31522, 34586, 34741, 35430, 35512, 37374, 39053, 39065, 39142, 39737, 40635, 41567, 41773, 41814, 42146, 42253, 43304, 43358, 45344, 45354, 45604, 47162, 47231, 47278, 47498, 49259, 49998, 50272, 50454, 50953, 51050, 51172, 51512, 51671, 52036, 52300, 52442, 53059, 53107, 53193, 53296, 53615, 54846, 55114, 55660, 55874, 57221, 58360, 58470, 58682, 58874, 59434, 59729, 60578, 60797, 61185, 61209, 61436, 61753, 62383, 62424, 62732, 62908, 63412, 63661, 64308, 64985, 65373, 65682, 66351, 67117, 68101, 68425, 69287, 69315, 69397, 71849, 72068, 73317, 73399, 73477, 76631, 76745, 77337, 77435, 77598, 77989, 78547, 78552, 78614, 80557, 81289, 81524, 81562, 82113, 82213, 82435, 83409, 83683, 84064, 84256, 84504, 84845, 86115, 86637, 87846, 88445, 88464, 88853, 89107, 89435, 89894, 90059, 90674, 90681, 90699, 92288, 92393, 92440, 93269, 93488, 93609, 94304, 95187, 95644, 95959, 98528, 98624, 99085, 99330, 99462. From Cleveland: 2480, 7835, 12330, 13495, 17946, 18011, 18566, 19005, 20003, 22736, 22935, 26557, 28596, 34189, 46075, 46739, 47482, 50092, 58811, 61868, 64706, 65858, 67936, 75398, 76139, 80263, 82518, 85091, 92232, 97844, 99664. From ICBM: 448, 5208, 10582, 16607, 26183, 28808, 32549, 35262, 40217, 44077, 49215, 55114, 59914, 68850, 77431, 85442, 87217, 93262, 94945, 98802. From Leiden: 1553, 1787, 4484, 8518, 9796, 10481, 12255, 13537, 18456, 19281, 28473, 30943, 36743, 38454, 39335, 40907, 52853, 52922, 56299, 57187, 58194, 64642, 66131, 68050, 72247, 86034, 87320, 92061, 93194, 97690, 99856. From Milwaukee: 917, 9931, 14692, 16666, 17004, 17987, 18955, 21350, 23607, 24237, 28782, 30157, 36386, 39259, 44912, 45019, 45852, 46312, 49975, 50771, 51182, 53971, 55176, 56084, 56108, 56333, 56582, 58677, 58967, 59359, 61779, 63196, 64463, 67948, 73547, 75919, 76042, 76378, 77073, 84314, 87784, 87910, 91468, 93170, 98971, 99479, . From Munich: 9035, 26223, 26670, 28697, 28902, 31272, 36052, 50162, 64202, 66933, 70942, 72095, 74607, 81737, 96591, 96752. From Newark: 13411, 32580, 36023, 41006, 43517, 49705, 53422, 54933, 55760, 58526, 59397, 59799, 62985, 71042, 71743, 76647, 78225, 86204. From New York: 1912, 2503, 3951, 4856, 5208, 7578, 8595, 9539, 10011, 10582, 12486, 13384, 14299, 14465, 15213, 15758, 16607, 17078, 17109, 18638, 19579, 20676, 20691, 20732, 21212, 22349, 22608, 23844, 24528, 26267, 27123, 28795, 28808, 29216, 29353, 29935, 30247, 30623, 30860, 31554, 31671, 33062, 33581, 35262, 37864, 38088, 41546, 44395, 44515, 44979, 45217, 46856, 47087, 47633, 48803, 48830, 50559, 51309, 51677, 53461, 53710, 54828, 56734, 59796, 63915, 69779, 73035, 77203, 77903, 84371.

**Dataset 4:** From *The Information eXtraction from Images study*, URL: ‑[http://brain‑development.org/ixi-dataset/](http://braindevelopment.org/ixi-dataset/), 563 subjects with the following IDs were used: 002, 012, 013, 014, 015, 016, 017, 019, 020, 021, 022, 023, 024, 025, 026, 027, 028, 029, 030, 031, 033, 034, 035, 036, 037, 038, 039, 040, 041, 042, 043, 044, 045, 046, 048, 049, 050, 051, 052, 053, 054, 055, 056, 057, 058, 059, 060, 061, 062, 063, 064, 065, 066, 067, 068, 069, 070, 071, 072, 073, 074, 075, 076, 077, 078, 079, 080, 083, 084, 085, 086, 087, 089, 090, 091, 092, 093, 094, 095, 096, 097, 098, 099, 100, 101, 102, 103, 104, 105, 106, 107, 108, 109, 110, 111, 112, 113, 114, 115, 116, 118, 119, 120, 121, 122, 123, 126, 127, 128, 129, 130, 131, 132, 134, 135, 136, 137, 138, 139, 140, 141, 142, 143, 144, 145, 146, 148, 150, 151, 153, 154, 156, 157, 158, 159, 160, 161, 162, 163, 164, 165, 166, 167, 168, 169, 170, 172, 173, 174, 175, 176, 177, 178, 179, 180, 181, 182, 183, 184, 185, 186, 188, 189, 191, 192, 193, 194, 195, 196, 197, 198, 199, 200, 201, 202, 204, 205, 206, 207, 208, 209, 210, 211, 212, 213, 214, 216, 217, 218, 219, 221, 222, 223, 224, 225, 226, 227, 229, 230, 231, 232, 233, 234, 236, 237, 238, 239, 240, 241, 242, 244, 246, 247, 248, 249, 250, 251, 252, 253, 254, 255, 256, 257, 258, 259, 260, 261, 262, 263, 264, 265, 266, 267, 268, 269, 270, 274, 275, 276, 277, 278, 279, 280, 282, 284, 285, 286, 287, 288, 289, 290, 291, 292, 293, 294, 295, 296, 297, 298, 299, 300, 302, 303, 304, 305, 306, 307, 308, 309, 310, 311, 312, 313, 314, 315, 316, 317, 318, 319, 320, 321, 322, 324, 325, 326, 327, 328, 329, 330, 331, 332, 334, 335, 336, 338, 342, 344, 348, 350, 351, 353, 354, 356, 357, 358, 359, 360, 361, 362, 363, 364, 365, 367, 368, 369, 370, 371, 372, 373, 375, 376, 377, 378, 379, 380, 381, 382, 383, 384, 385, 386, 387, 388, 389, 390, 391, 392, 393, 394, 395, 396, 397, 398, 399, 400, 401, 402, 403, 404, 405, 406, 407, 408, 409, 410, 411, 412, 413, 414, 415, 416, 417, 418, 419, 420, 422, 423, 424, 425, 426, 427, 428, 429, 430, 431, 432, 433, 434, 436, 437, 438, 439, 440, 441, 442, 443, 444, 445, 446, 447, 448, 449, 450, 451, 452, 453, 454, 455, 456, 458, 459, 460, 461, 462, 463, 464, 465, 467, 468, 469, 470, 473, 474, 475, 476, 477, 478, 479, 480, 481, 482, 483, 484, 485, 486, 487, 488, 489, 490, 491, 492, 493, 494, 495, 496, 497, 498, 499, 500, 501, 502, 503, 504, 505, 506, 507, 508, 510, 511, 512, 515, 516, 517, 518, 519, 521, 522, 523, 524, 525, 526, 527, 528, 531, 532, 533, 534, 535, 536, 537, 538, 539, 541, 542, 543, 544, 546, 547, 548, 549, 550, 551, 552, 553, 554, 555, 556, 558, 559, 560, 561, 562, 563, 565, 566, 567, 568, 569, 571, 572, 573, 574, 575, 576, 577, 578, 579, 582, 584, 585, 586, 587, 588, 591, 592, 593, 594, 595, 596, 597, 598, 599, 600, 601, 603, 605, 606, 607, 608, 609, 610, 611, 612, 613, 614, 616, 617, 618, 619, 621, 622, 625, 626, 627, 629, 630, 631, 632, 633, 634, 635, 636, 639, 640, 641, 642, 644, 646, 648, 651, 652, 653, 662.
